# Triple role of exosomes in lung transplantation

**DOI:** 10.3389/fimmu.2025.1544960

**Published:** 2025-04-11

**Authors:** Dingyu Rao, Defa Huang, Zongbo Peng, Dewang Xiao, Chunfa Xie, Shenyu Zhu, Haoquan He, Zhixian Tang, Zhongkai Wu, Zuxiong Zhang

**Affiliations:** ^1^ Department of Thoracic Surgery, First Affiliated Hospital of Gannan Medical University, Ganzhou, China; ^2^ Laboratory Medicine, The First Affiliated Hospital of Gannan Medical University, Ganzhou, China; ^3^ The First Clinical College, Gannan Medical University, Ganzhou, China; ^4^ Department of General Practice, Jinhua Jindong District Xiaoshun Town Ditian Community Health Centre, Jinhua, China; ^5^ Department of Cardiac Surgery, First Affiliated Hospital of Sun Yat-Sen University, Guangzhou, China

**Keywords:** exosome, lung transplantation, DCs, biomark assay, allograft immunization

## Abstract

Exosomes are tiny vesicles secreted by the vast majority of cells and play an important role in physiological as well as pathological processes in the body. Circulating exosomes in Lung Transplant Recipients (LTxR) undergoing rejection contain mismatched Human Leukocyte Antigens (HLA) and lung-associated autoantigens (e.g., K-alpha1 microtubule protein and collagen V), which may induce autoantibodies, and the circulating exosomes trigger an immune response that results in rejection of the lung transplant recipient. This article discusses the role of exosomes in lung transplantation from three perspectives: exosomes as a biomarker for rejection after lung transplantation; the mechanism of exosome-mediated activation of the immune response; and the potential of exosomes as a therapeutic strategy.

## 1 Introduction

Organ transplantation is the most effective treatment for end-stage organ failure, and the establishment of organ transplantation technology is one of the most important advances in human biomedicine in the 20th century. Lung transplantation is the only effective treatment for end-stage lung disease (1–3). Since 1990, more than 70,000 cases have been completed worldwide (4). In recent years, lung transplantation (LTx) technology has been developing rapidly, but at the same time, it faces many obstacles. Basic research on transplantation immunology is a necessary way to solve the rejection reaction of organ transplantation and a source of driving force for the development of technological innovation related to organ transplantation. With the continuous development of medical technology and immunosuppression, LTx has become more and more mature, but there are still many postoperative complications, such as infection, acute rejection reaction (AR), chronic rejection reaction (CR), acute pulmonary edema, and cardiovascular disease, etc. Among them, CR is the most common after LTx, with an incidence of about 50% within 5 years and a rate of up to 90% within 10 years (5).

Exosomes are nanovesicles with an average size of 40-100 nm. They contain membrane and cytoplasmic proteins, as well as molecules essential for exosome biogenesis, and are secreted by a variety of cells and released in biological fluids (6). The composition of exosomes is highly dependent on the biological function of the parental cells. Thus, exosomes contain specific microRNAs (miRNAs), mRNAs, and proteins associated with specific cell types, including epithelial cells, B cells, T cells, and Dendritic Cells (DCs) (7–9).

Exosomes are involved in physiological, (e.g., embryo implantation, regulatory functions of semen, pregnancy, regulation of immune responses) and pathological processes such as cancer, development of neurodegenerative diseases (8, 10). They are also involved in rejection/tolerance of recipient kidney transplants (11, 12) as well as in the pathogenesis of autoimmune diseases, including rheumatoid arthritis, desiccation syndrome and systemic lupus erythematosus. Exosomes can functionally transfer their cargoes such as mRNAs and miRNAs to recipient cells, Thus, with respect to the transfer of cargo and its effects on other cells, exosomes can be considered carriers of cell-cell communication, just like other types of cell-cell communication (including secretion of soluble factors [e.g., cytokines and chemokines], as well as cell-contact-dependent communication [e.g., cytosolic nibbling, membrane nanotubes, or nibbling]) (13, 14). Exosomes use different mechanisms to carry out their pathologic actions. In the case of transplantation, exosomes participate in the activation or suppression of the immune response by presenting allogeneic MHC peptides to allogeneic-specific T cells.

Based on the above characteristics, exosomes play an important role in immune activation or suppression of allograft immunization. The presence of alloantigens, cell-specific antigens, peptides, and co-stimulatory molecules on the surface of exosomes, as well as the presence of nucleic acids, lipids, small RNAs, and transcription factors inside exosomes that are released after transplantation, make exosomes one of the attractive targets for the identification of biomarkers related to allogeneic transplantation immunity. Circulating exosomes in Lung Transplant Recipients (LTxRs) are characterized by the presence of tissue-associated Self-Antigens (SAgs)-K-α1 Tubulin (Ka1T) and Collagen V (Col-V), costimulatory molecules, the transcription factor Nuclear Factor κB (NF-κB), Hypoxia-Inducible Factor (HIF), 20S proteasome, MHCP, and other factors, MHC class II molecules and its transcription factor CIITA (15). By studying the biological components of exosomes, it is crucial to understand their role in transplant rejection. Sigdel et al. (16) demonstrated that urinary exosomes isolated from acutely rejected kidney transplant recipients (KTxR) contained proteins associated with inflammatory responses; urinary exosomes isolated from stable KTxR did not. A recent report by Dieude et al. (17) also showed that exosomes isolated from HUVEC and mouse endothelial cells contain active 20S proteasomes that increase the immunogenicity of the exosomes, leading to the production of antibodies against renal-associated SAg bead proteoglycans in a mouse model. Different cargoes are transferred between different cell populations and have different roles.

**Figure 1:**
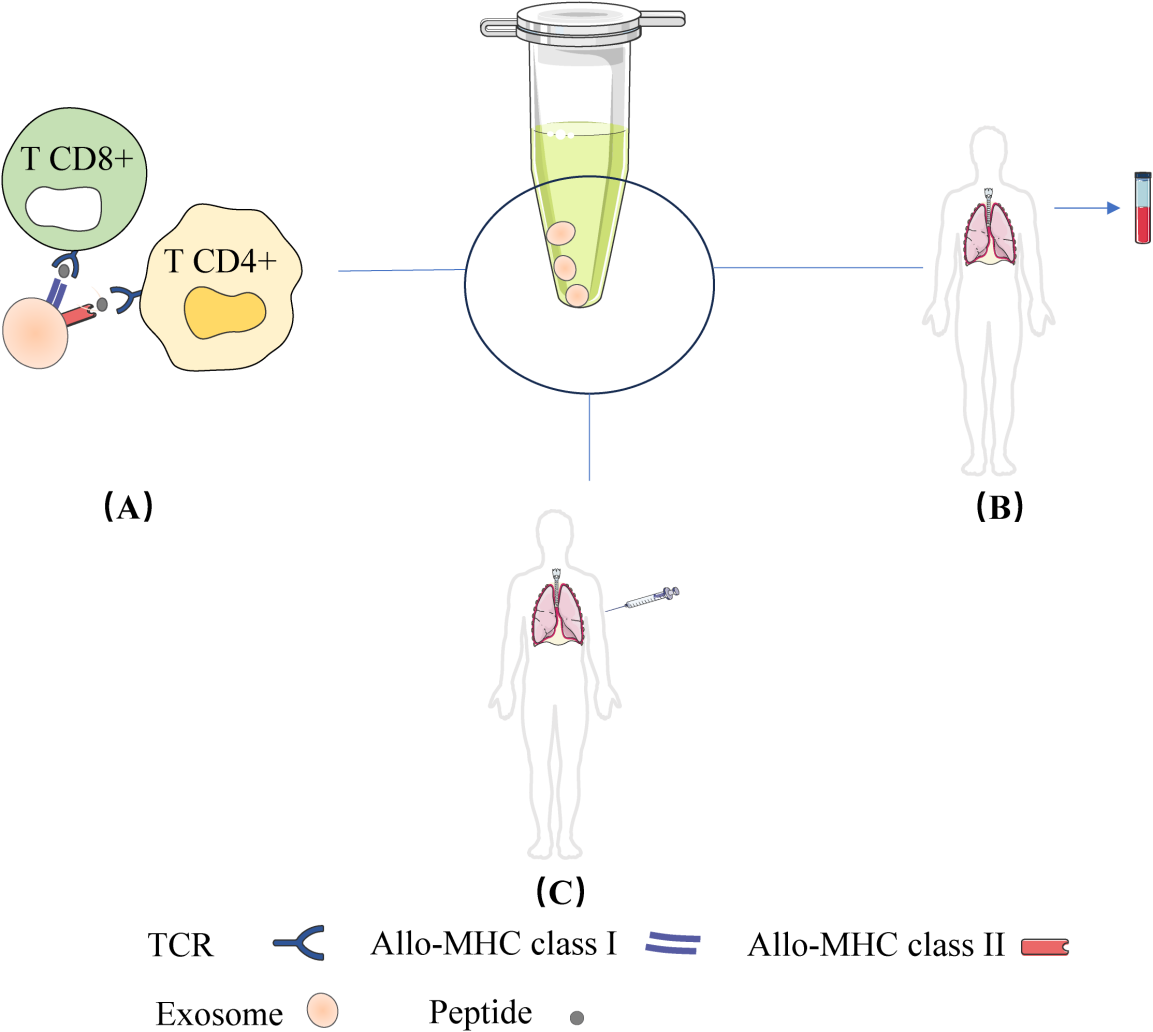
Three roles of the exosomes in transplantation recipients or in transplantation models: **(A)** Role as Antigen-Presenting Vesicles; **(B)** Role as as biomarkers; **(C)** Role as therapeutic strategies.

In this review, we will explore recent advances in exosomes in terms of isolation, characterization techniques and half-life, explaining in detail the different types of exosome-mediated allograft immunization, emphasize the value of exosomes released during post-transplant rejection as biomarkers and therapeutic targets, review and discuss the triple roles of exosomes in transplantation recipients or in transplantation models (role as Antigen-Presenting Vesicles (APVs), role as biomarkers, and role as therapeutic strategies) in order to elucidate their role in the field of transplantation[as shown in **Figure 1**].

## 2 Current status and technological advances in exosomal research: isolation, identification and half-life

The exosomes contain a variety of biologically active substances, such as proteins, lipids and nucleic acids, and these components not only reflect the physiological state of the mother cell, but also influence the progression of the disease by regulating the function of the target cells (18).

### 2.1 Gold standard techniques in exosome research

Exosome research has gained significant attention due to the critical role these extracellular vesicles play in intercellular communication and their potential as biomarkers for various diseases. The standard techniques for isolating and characterizing exosomes are crucial for advancing this field. The gold standard methods include ultracentrifugation, immunoaffinity methods, and commercially available separation kits. Each of these techniques has its advantages and limitations, and their effectiveness can vary based on the specific application and sample type (19) variations in protocols, such as the number of centrifugation cycles and the use of sucrose gradients, can impact the purity and yield of isolated exosomes (20).

### 2.2 Best separation methods for exosomes

The isolation of exosomes is a critical step in understanding their biological functions and potential therapeutic applications. Various methods have been developed to separate exosomes from biological fluids, each with its advantages and limitations. The choice of separation method can significantly influence the yield, purity, and functionality of the isolated exosomes. Among the most commonly used techniques are ultracentrifugation, size exclusion chromatography, and immunoaffinity capture. Ultracentrifugation, while being the traditional gold standard for exosome isolation, can be time-consuming and may result in the co-isolation of non-exosomal contaminants. Size exclusion chromatography offers a more gentle approach, allowing for the separation of exosomes based on size, thus minimizing damage to their structure. Immunoaffinity capture utilizes antibodies specific to exosomal surface markers, providing high specificity but requiring prior knowledge of the markers present on the exosomes of interest (21). Recent advancements in microfluidic technologies and novel separation techniques continue to enhance the efficiency and specificity of exosome isolation (22), paving the way for their application in diagnostics and therapeutics (23).

### 2.3 Description of common methods for exosome characterization

Nanoparticle Tracking Analysis (NTA) is a widely utilized technique for characterizing exosomes based on their size and concentration. NTA employs laser light scattering to track the Brownian motion of individual nanoparticles, allowing for real-time measurement of their size distribution and concentration in a sample. The technique is particularly advantageous due to its ability to analyze particles in the size range of 40 to 1000 nm, which encompasses the typical size of exosomes (24). One of the key strengths of NTA is its capability to provide a comprehensive size distribution profile, which is critical for understanding the heterogeneity of exosome populations derived from different biological sources. Moreover, NTA is relatively quick and requires minimal sample preparation, making it suitable for high-throughput applications (25). However, NTA also has limitations, including sensitivity to sample concentration and the potential for inaccuracies in size estimation due to the presence of aggregates or contaminants (26). Despite these challenges, NTA remains a valuable tool for exosome characterization, particularly when combined with other analytical techniques to enhance the robustness of findings.

Transmission Electron Microscopy (TEM) is a powerful imaging technique that allows for the visualization of exosomes at the nanoscale. TEM provides detailed morphological information about exosomes, including their size, shape, and structural integrity, which are crucial for understanding their biological roles (27). The method involves the transmission of electrons through ultra-thin sections of exosome preparations, yielding high-resolution images that can reveal the presence of specific surface markers through immunogold labeling techniques (28). TEM is particularly beneficial for examining the ultrastructure of exosomes and assessing their purity, as it enables researchers to distinguish exosomes from other extracellular vesicles and contaminants (28). However, the application of TEM is limited by its requirement for extensive sample preparation, which can introduce artifacts and may alter the native state of exosomes (27). Additionally, TEM is not suitable for quantitative analysis of exosome populations, making it essential to complement this method with quantitative techniques like NTA or proteomic analysis for a comprehensive understanding of exosome characteristics.

Proteomic analysis is a critical method for characterizing the protein composition of exosomes, providing insights into their functional roles and potential therapeutic applications. This technique involves the identification and quantification of proteins present in exosomes using Mass Spectrometry (MS), which has become the gold standard for proteomic studies (29). Proteomic profiling of exosomes can reveal distinct protein signatures that correlate with specific biological processes or disease states, thereby aiding in the identification of potential biomarkers for diagnostics or therapeutic targets (30). High-throughput proteomic technologies have advanced significantly, allowing for comprehensive analysis of exosomal proteins, including post-translational modifications that may influence their function (31). However, challenges remain in standardizing sample preparation methods and ensuring reproducibility across studies, particularly given the complexity and heterogeneity of exosomal samples (32). Despite these challenges, proteomic analysis is an invaluable tool for elucidating the biological roles of exosomes and advancing their application in clinical settings.

In conclusion, each of these methods—NTA, TEM, and proteomic analysis—offers unique advantages for the characterization of exosomes. A comprehensive characterization approach that integrates these techniques can enhance our understanding of exosome biology and facilitate their application in diagnostics and therapeutics.

### 2.4 Exosome surface markers and internal protein identification techniques

Western blotting remains a foundational technique for protein analysis, allowing for the detection and quantification of specific proteins in exosome samples. This method involves the separation of proteins by gel electrophoresis, followed by transfer to a membrane where they can be probed with specific antibodies. The sensitivity and specificity of Western blotting make it particularly suitable for identifying exosomal proteins, as it can detect low-abundance proteins that may play critical roles in disease processes (33).

However, the technique does have limitations, including the potential for cross-reactivity and the need for high-quality antibodies. Recent advancements have sought to improve the efficiency of Western blotting, such as optimizing antibody concentrations and using total protein normalization methods to enhance quantification accuracy (34). Additionally, novel strategies, such as the use of aptamers instead of antibodies, have been explored to provide a more cost-effective and efficient approach to protein detection (35). Overall, Western blotting continues to be a valuable tool for exosome research, particularly in the context of identifying disease-associated proteins.

Flow cytometry is a powerful technique that allows for the rapid analysis of exosome populations based on their size and surface markers. This method utilizes laser technology to analyze individual exosomes as they pass through a detection point, providing information on their physical and chemical properties. With the ability to simultaneously measure multiple parameters, flow cytometry can identify specific exosome subpopulations based on the expression of surface proteins, which is particularly useful for understanding their roles in various biological processes (36). The use of fluorescently labeled antibodies enables the quantification of exosome surface markers, facilitating the identification of exosomes derived from specific cell types or disease states. Recent innovations in imaging flow cytometry have further enhanced the capabilities of this technique, allowing for the visualization of exosomes while maintaining high throughput (37). However, challenges remain, such as the need for standardized protocols to ensure reproducibility and the potential for signal overlap when analyzing multiple markers. Despite these challenges, flow cytometry remains an essential tool for exosome characterization and has significant implications for diagnostics and therapeutic monitoring.

Mass spectrometry (MS) has emerged as a critical technique for the comprehensive analysis of exosomal proteins and their molecular constituents. This method provides high sensitivity and specificity, allowing for the identification and quantification of proteins, lipids, and nucleic acids within exosomes (38). Mass spectrometry can be combined with various sample preparation techniques, such as liquid chromatography, to enhance the separation and identification of complex mixtures (39). The ability to analyze post-translational modifications and protein interactions makes mass spectrometry particularly valuable for understanding the functional roles of exosomal proteins in cellular communication and disease progression. Recent advancements in mass spectrometry technologies, including high-resolution mass analyzers and novel ionization techniques, have significantly improved the detection limits and accuracy of exosome analysis (40). However, challenges such as sample complexity and the need for robust bioinformatics tools for data interpretation remain. Overall, mass spectrometry represents a powerful approach for elucidating the molecular profiles of exosomes, providing insights into their biological significance and potential as therapeutic targets.

### 2.5 Half-life of exosomal markers

Understanding the *in vivo* half-life of exosomes is critical for their application in transplant immunology, particularly regarding their stability and efficacy as therapeutic agents. Studies have demonstrated that exosomes can exhibit variable half-lives depending on their origin and the biological environment (41). For instance, exosomes derived from mesenchymal stem cells (MSCs) have shown prolonged circulation times *in vivo*, which enhances their potential as therapeutic agents for promoting graft survival and tissue repair. The half-life of exosomes can be influenced by factors such as their size, surface charge, and the presence of specific surface proteins that facilitate interactions with recipient cells (26). Moreover, engineering exosomes to improve their stability and targeting capabilities is an area of active research, with the aim of enhancing their therapeutic efficacy in transplant settings.

## 3 Role of exosomes in allograft immunization

**Figure 2:**
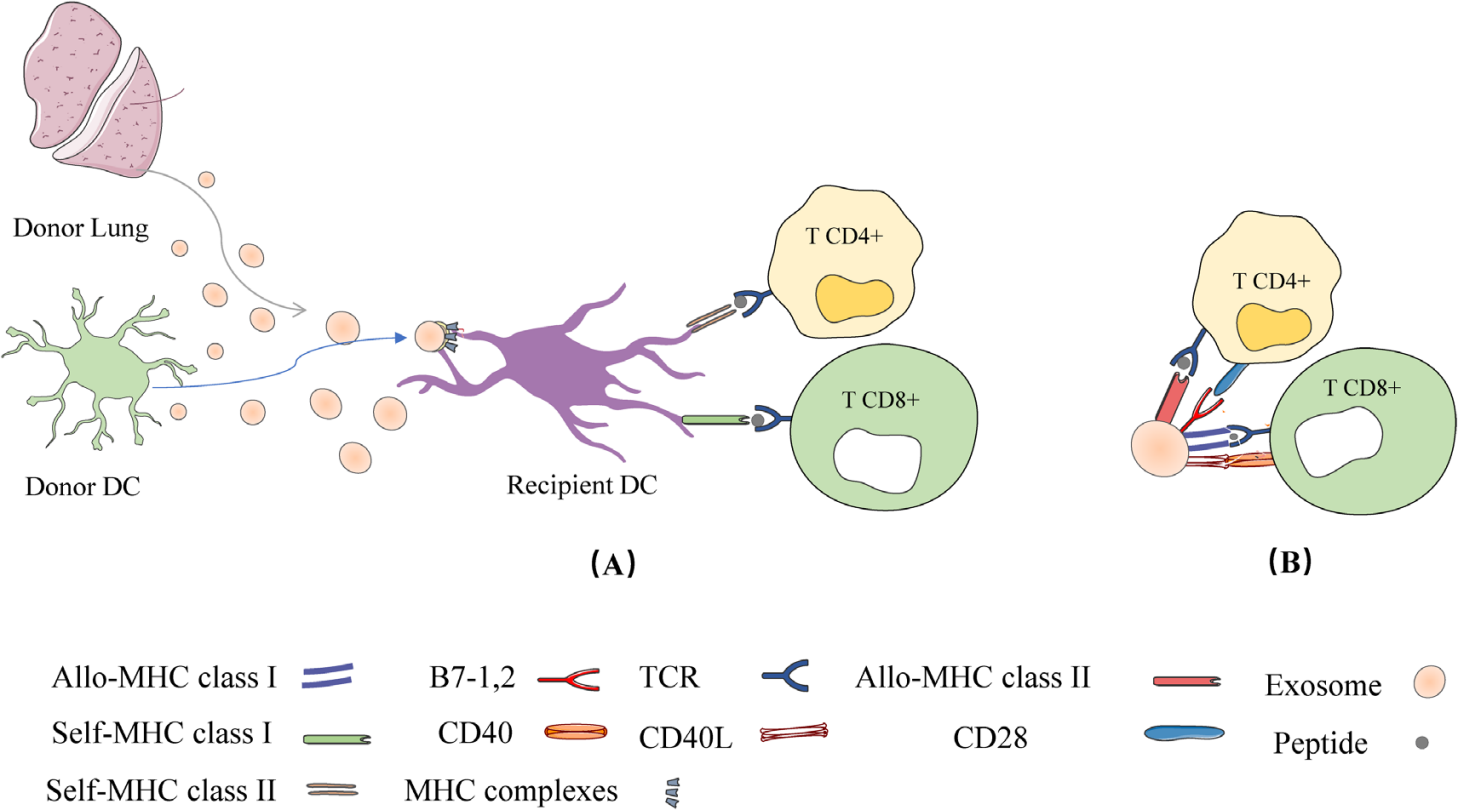
Exosome mediated allogeneic recognition of T cells in local lymph nodes: **(A)** Exosomes released from allogeneic grafts or donor DCs are captured by recipient DCs and their cognate MHC is expressed on the surface of the recipient DCs (called cross-modified cells). The cross-modified cells present the cognate MHC peptide to T cells; **(B)** In addition to APC, exosomes secreted by allogeneic grafts or donor DCs can directly present their allo-MHC-peptides to T cells.

The primary cause of allogeneic graft rejection is through allogeneic immune responses mediated by T cells, B cells, macrophages, dendritic cells (DCs), and others. These pathways include direct, indirect, and semi-direct allograft recognition (42, 43). In the direct pathway, donor DC migrates to regional lymph nodes (LNs) and presents whole allogeneic Major Histocompatibility Complex (MHC) peptides to recipient T cells, whereas in the indirect pathway, recipient DC captures allogeneic antigens (e.g., isoallogeneic MHC) and presents them to allogeneic T cells in the context of their own MHC. By an indirect pathway, the alloantigen is internalized by the receptor APC, processed by its own MHC class II molecules and presented as a peptide to CD4+ T cells [as shown in **Figure 2**]. This mechanism has been described to persist throughout the allograft period and is considered the primary cause of chronic graft rejection, which is dominated by the production of CD4 + T cells and alloreactive antibodies. Patients with occlusive Bronchiolitis Obliterans (BO) have a significantly higher frequency of indirect donor allogeneic reactions than patients without BO (44, 45). The researchers later found that direct alloreactive T cells also recognize intact donor MHC molecules acquired by acceptor APCs through a third mechanism, the semi-direct pathway. The semi-direct pathway has also been used to explain the cross-regulation between CD4 and CD8 T cells activated by different pathways (46, 47). The mechanisms by which recipient APCs acquire and retain fully functional donor MHC molecules in the semi-direct pathway are unknown. New evidence from the field of extracellular vesicles, especially exosomes, is now providing new answers to these long-standing questions in transplantation and allogeneic recognition.

Secreted by a variety of APCs, including mast cells, natural killer cells, Dendritic Cells (DCs), macrophages, T cells, and B cells, the exosomal surface MHC peptide complexes are highly enriched, suggesting that they can act as antigen-presenting vesicles or vectors to disseminate allogeneic antigens to initiate anti-donor T cells (48, 49). Exosomes produced by DCs have been observed in primary DCs and DC cell lines and have been the focus of many studies in the field of exosome-mediated allosome recognition. Mature dendritic cells release exosomes enriched for MHC, adhesion and T-cell co-stimulatory molecules, mature dendritic cells also encounter secreted exosomes from a variety of other cellular subpopulations and present ectoparasitic antigens to stimulate or inhibit cellular (50). Montecalvo and his colleagues described a short-range mechanism for the distribution of alloantigens to the dc via an exosomal delivery mechanism, leading to the triggering of the anti-donor T response observed in transplantation. It was also shown that exosomes are more important than soluble isopeptides in the transfer of antigens to the dc, because antigens internalized and secreted as phagosomes are 103- 104 times more efficiently presented in MHC II (50–52). In addition, Sureshbabu et al. (53) induced Obstructive Airway Disease (OAD), similar to human CLAD, by intra-bronchial administration of MHC antibodies in mice, and importantly, antibodies to lung-associated SAgs (Col-V, Kα1T) on exosomes were detected before the clinical diagnosis of OAD, demonstrating that exosomes activate the immune response, leading to the production of antibodies to lung SAgs and ultimately lead to the development of OAD. Clearance of donor leukocytes, or the inability to migrate to recipient lymphoid tissue, suggests that exosomes of donor graft origin play an important role in the alloantigen recognition pathway.

### 3.1 The role of exosomes in direct/indirect recognition

**Figure 3:**
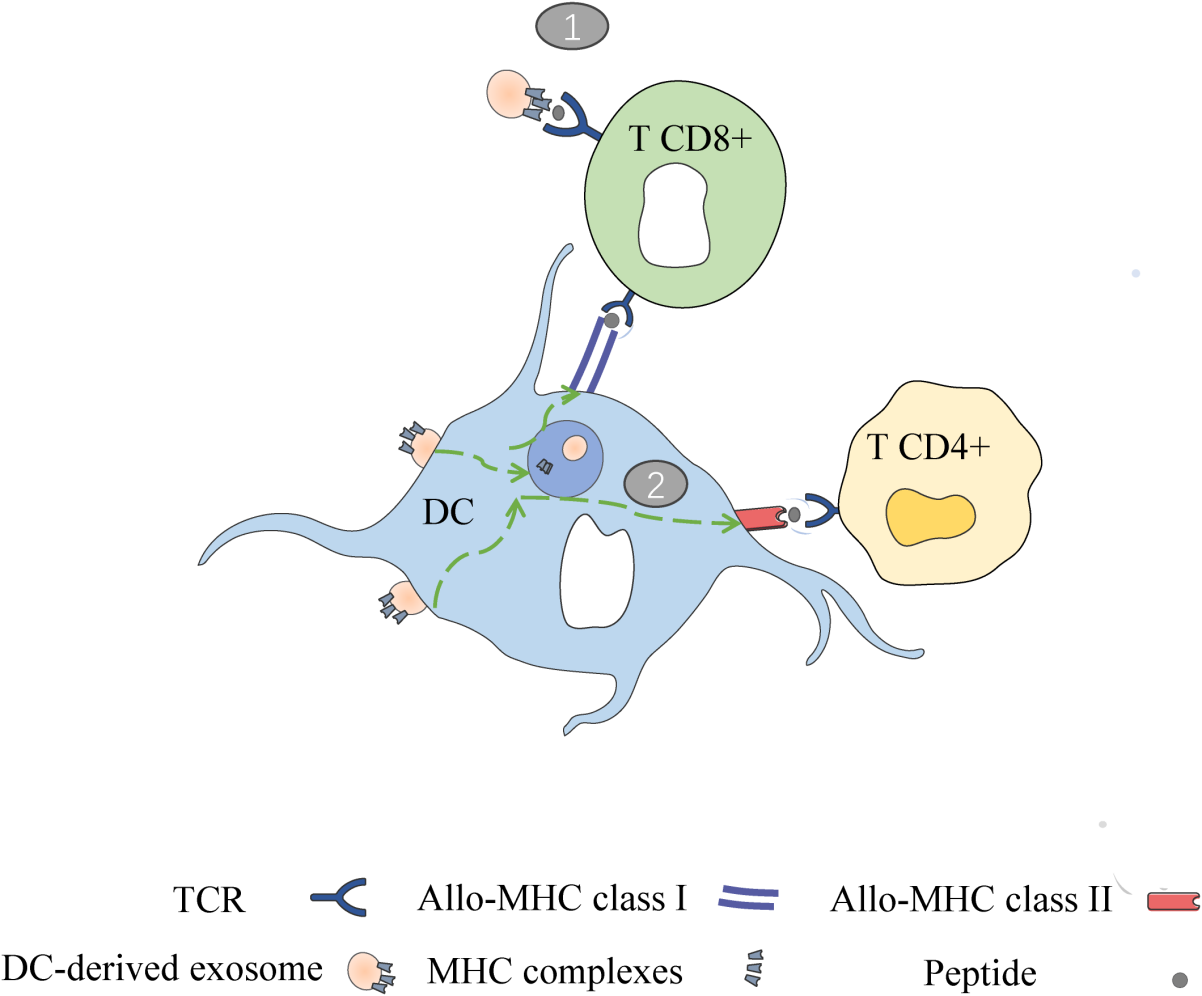
Two ways of DC derived exosomes (DC-EXO) mediated antigen presentation. (1) DC-EXO can directly present antigen to T cells and stimulate T cell activation; (2) After binding to APCs, DC-EXO merges with the acceptor APC surface membrane and transfers its peptide/MHC complexes. Following internalization, the DC-EXO peptide/MHC complexes can be reprocessed via endosomal pathways within the APC. Peptide complexes can then be transported back to the DC’s surface for presentation to T cells.

Peche et al. (2006) as well as Segura et al. (2005) demonstrated a direct role of exosomes in the induction or suppression of immune responses in the context of transplantation. In other words, they noted that in addition to APCs, exosomes can present their MHC peptides to T cells and activate them (51, 54). However, when exosomes are captured by DCs, this leads to effective T cell stimulation and therefore requires APCs to present allo-MHC-peptides derived from exosomes to T cells (semi-direct pathway) (50, 55). In the direct pathway, DC-derived exosomes can directly present their MHC peptides to T cells. In the semi-direct pathway, DC-derived exosomes can be captured by receptor DCs so that their entire MHC peptide can be presented to T cells via DCs, which is referred to as the heterodimerization phenomenon [as shown in **Figure 3**] (56).

In the indirect pathway, although free exosomes have a limited ability to directly induce allogeneic reactive T cells, they can act as a peptide source to indirectly initiate T cells when the p-MHC complexes they carry are internalized by APC through phagocytosis or microcellular drinking (57). Robbins and morelli demonstrated that exosomes carrying homodimeric peptides can trigger specific cd4T cell responses in wild-type mice, but not in MHC class II-deficient hosts (58).

### 3.2 The role of exosomes in semi-direct recognition

A recent study has shown, based on ultrastructural observations, that exosomes are involved in semi-direct pathways (59). After skin and heart allografts, DCs and B cells in the graft-draining lymphoid organs are cross-modified with donor-derived exosomes carrying intact donor MHC molecules (59, 60). Notably, rather than being internalized by recipient DCs as previously described, exosomes adhered to the surface of recipient DCs in small clusters, retaining intact and functional donor MHC molecules and APC activation signals. Depletion of these DCs greatly diminished T-cell sensitization to donor MHC and delayed cardiac allograft rejection (60). Since indirect allogeneic-reactive T helper cells and direct allogeneic-reactive T effector cells are differentially affected by host DCs after exposure to “heterodimerization” exosomes, this process was observed during chronic allogeneic-exosome exposure (61).

Exosomes are not only involved in the three pathways of allogeneic recognition, but also activate the immune system by presenting their mhc peptides to T cells other than APC. In addition, there are no reliable non-invasive biomarkers to monitor early post-transplant status. Such biomarkers may be important for graft management and to improve long-term survival of allogeneic grafts. Since exosomal levels and surface markers are significantly different in rejection and non-rejection patients, they can be considered as new predictive or diagnostic biomarkers in the field of transplantation.

## 4 Exosomes as biomarkers of allograft rejection

After LTx, clinicians monitor rejection by methods such as bronchoscopic biopsy and imaging. However, these methods lack accuracy and specificity and often reflect non-early stages of graft lung injury. Therefore, there is an urgent need to develop new diagnostic biomarkers in the field of LTx for noninvasive and continuous monitoring of allogeneic LTx. LTxRs exosomes, which differ in their major components in the presence or absence of rejection, hold promise as predictive/diagnostic biomarkers of allograft rejection.

### 4.1 Role of exosomal proteins in biomarkers

As medical technology and immunosuppression continue to evolve, LTx is becoming more and more sophisticated, However, there are still many postoperative complications, such as infection, Acute Rejection (AR), Chronic Rejection (CR), acute pulmonary edema, and cardiovascular disease. Among them, CR is the most common after LTx, with an incidence of about 50% within 5 years and up to 90% within 10 years (62). Lung failure due to Chronic Lung Allograft Dysfunction (CLAD) in CR is the leading cause of death within one year of transplantation (63). CLAD consists of two subtypes: obliterative (BOS) and restrictive allograft syndrome (RAS).BOS is the most common clinical manifestation of CLAD and occurs in approximately 70% of patients with CLAD (53).

The main reason for rejection is the mismatch between the allograft and the recipient’s Human Leukocyte Antigen (HLA), which is recognized by the recipient’s immune monitoring. Sureshbabu et al. (53) reported the presence of exosomes in the serum and BALF of LTxRs diagnosed with AR and CR, which carry specific antigens and are involved in the rejection of allogeneic LTx. Circulating exosomes are induced by Primary Graft Dysfunction (PGD), ischemia-reperfusion injury, Respiratory Viral Infection (RVI), and the production of Donor-specific antibodies (DSA) and antibodies to lung-associated SAgs (Ka1T and Col-V) (64). Circulating exosomal expression in CLAD patients: recipient mismatched HLA molecules, lung-associated SAgs, co-stimulatory molecules (CD80, CD86), transcription factors NF-κβ, HIF-1α, microRNAs (miRNAs), and 20S proteasome (15). Gunasekaran et al. (65) found that exosomes from non-rejected patients do not express MHC class II molecules and co-stimulatory molecules (CD80, CD86, CD40), but adhesion molecules are expressed on circulating exosomes from LTxRs.

The production of antibodies to HLA molecules and/or lung-associated SAgs is a predictive marker of rejection. In LTxRs in which rejection occurred, donor HLA and SAgs were detected on the surface of donor-sourced exosomes, and no SAgs were detected on donor-sourced exosomes from stable LTxRs, suggesting that circulating exosomes originated from transplanted organs after immune injury (53). Exosomes of donor origin “leak” out of the graft and flow through extravasated or severed openings in the recipient’s capillary lymphatics to the lymphoid organs draining the graft. Habertheuer et al. (66) transplanted left lungs from Wistar transgenic mice into Lewis recipients with complete MHC molecular mismatch, and GFP-labeled CD63 was used to detect exosomes levels, with circulating exosomes peaking on day 1, decreasing significantly on day 2, and then reaching baseline levels on day 3, consistent with the manifestation of AR. The rapid decline in exosome levels, occurring before histologic evidence of AR in the graft, suggests that exosomes may serve as a new biomarker.

Rahman et al. (64) observed in a mouse model of *in situ* unilateral LTx CR that more than 80% of transplanted mice showed elevated levels of lung SAgs (Col-V and Ka1T) by exosomes isolated from serum from day 14 onwards, and also the presence of anti-lung SAgs antibodies in the serum, whereas histological changes of CR appeared only on day 30 onwards, suggesting that exosomes may be a potential CR biomarkers and hypothesized that exosomes may be involved in the development of CR after LTx.

Sharma et al. (67) showed that exosomes isolated from plasma 12 months prior to the clinical diagnosis of BOS showed elevated levels of pulmonary SAgs (Ka1T and Col-V) (specificity 100%, sensitivity 90%), suggesting that circulating exosomes with pulmonary SAgs can be used as a biomarker to identify LTxRs at risk of BOS.

The Clavate Cell Secreted Protein (CCSP) has anti-inflammatory properties, and levels of CCSP are decreased in smoking, infections, lung injury, BOS, and other diseases that can lead to an inflammatory response.

According to Itabashi et al (68), CCSP levels in the BALF of patients were significantly decreased 7 to 9 months prior to the clinical diagnosis of BOS. Low levels of CCSP can promote the production of pro-inflammatory cytokines, induction of natural killer cell (NK cell)-derived exosomes, and immune responses to HLA and SAgs. NK cell-derived exosomes contain increased SAgs, NK cell markers, and cytotoxic molecules, suggesting that NK cell-derived exosomes play a role in the development of CLAD. The absence of CCSP leads to the release of exosomes from NK cells, which can stimulate intrinsic and adaptive immune responses after transplantation.

Goodlet et al. (69) reported that in a 76-year-old female lung transplant patient who received immunosuppressive therapy for AR and was subsequently infected with SARS-CoV-2, HLA antibody levels began to increase dramatically. Analysis of circulating exosomes prior to SARS-CoV-2 infection revealed the presence of pulmonary SAgs, HLA-DR, and HLA-DQ. After the patient was infected with SARS-CoV-2, exosomes were found to contain SARS-CoV-2 spiking proteins, and after the symptoms of the infection resolved, exosomes containing SARS-CoV-2 spiking proteins were no longer detected; however, exosomes with lung SAgs, HLA-DR, and HLA-DQ were consistently present, and lung function continued to decline, suggesting that the patient had CLAD. The above suggests that detection of exosomes containing viral proteins may be useful in recognizing allograft injury caused by viral infection.

Anti-HLA and anti-lung-associated SAgs may play a synergistic role in the immunopathogenesis of BOS and are important predictors of BOS progression (70). Most patients who develop DSA also subsequently develop lung-associated SAgs antibodies, suggesting that DSA may activate and induce lung-associated SAgs antibody production (71). An analysis of 103 LTxRs by Sureshbabu et al. (53) showed that 42.7% of LTxRs produced DSA and 30.1% produced Kα1T and ColV antibodies, suggesting that the production of DSA usually precedes the production of lung SAgs antibodies. In addition, HLA antigens and lung-associated SAgs, interacting with each other, had a greater probability of producing antibodies, suggesting the presence of immunodiffusion. It was shown that lung-associated SAg (Kα1T) induced not only antibodies against Kα1T, but also antibodies against Col-V, suggesting that spreading of the immune response occurs prior to BOS formation in the mouse LTx CR model. However, because HLA molecules and lung-associated SAgs are encoded by different genes on different chromosomes, the mechanisms that lead to their spread are unclear (15).

### 4.2 The role of exosomal nucleic acids in biomarkers

Micro-RNAs can be used as new diagnostic biomarkers to identify patients at risk of developing BOS. miRNAs are a hot topic of research in the field of organ transplantation, where they bind to target mRNAs to regulate the expression of genes that are key elements of the innate and adaptive immune response. The miRNAs are a hot topic in the field of organ transplantation. Exosomes are known to contain miRNAs that trigger immune responses by inducing inflammation, endothelial activation, Th17 differentiation, and antibody-mediated rejection. Exosomes interact with cells through ligand-receptor specific binding, fusing with the cytosol of target cells and “injecting” their inclusions into the cytoplasm of target cells. miRNAs in exosomes regulate the mNA of target cells (72). Xu et al. (73) showed that the expression levels of miR-134, miR-10a, miR-195 and miR-133b were significantly down-regulated, and miR-144, miR-142-5p and miR-155 expression were significantly up-regulated, compared to stable LTxRs serum samples at 12 months of BOS. LTxRs at risk for BOS development can be distinguished based on the above miRNAs.

Therefore, as shown in **Table 1**, it is believed that circulating exosomes with tissue specificity are expected to be a noninvasive biomarker that can monitor the risk of rejection in organ transplant recipients and provide great help in clinical transplantation.

**Table 1:** Characterization of exosomes from lung transplant patients with complications.

Study subjects	Sample	Species	Exosome content	References
CLAD	Serum	Human	Col-V, Ka1T	(15)
BOS	BALF	Human	CCSP	(81)
Rejected patients	Serum	Human	CD80, CD86,CD40	(78)
CLAD	Serum	Human	NF-KB, HIF-1a, 20S proteasome	(17)
BOS	Serum	Human	LKβ1	(75)
OAD	Serum	Mice	Col-V, Ka1T	(66)
CLAD	Serum	Human	HLA-DR, HLA-DQ	(82)

CLAD, Chronic Lung Allograft Dysfunction; BOS, Bronchiolitis Obliterans Syndrome; OAD, Obstructive Airway Disease; Col-V, Collagen V; Ka1T, Self-Antigens (SAgs)-K-α1 Tubulin; CCSP, Clavate Cell Secreted Protein; NF-KB, transcription factor Nuclear Factor κB; HIF-1a, Hypoxia-Inducible Factor-1a; LKβ1, Liver kinase β1; HLA-DR, Human Leukocyte Antigen-DR; HLA-DQ, Human Leukocyte Antigen-DQ.

## 5 Therapeutic strategies for exosomes in LTx rejection reactions

Exosomes are also potent candidates for targeted drug delivery and offer various advantages over traditional synthetic materials such as liposomes in drug delivery. The therapeutic potential of exosome-mediated drug delivery is still in preliminary clinical trials [pancreatic cancer (74, 75), acute ischemic stroke (76, 77) and colon cancer (78, 79)].

Several studies have shown that the pathophysiology of BOS involves inflammation, fibroblast proliferation; extracellular matrix deposition (80–82), and evidence of increased Epithelial-Mesenchymal Transition (EMT) markers in bronchial epithelial cells (62, 83).

Liver kinase β1 (LKβ1), also known as serine/threonine protein kinase 11, acts as a tumor suppressor in cancer (84–86). In a recent study, exosomes isolated by Rahman et al. (62) from the plasma of patients diagnosed with BOS or LTxRs with stable lung function, and induced morphological and molecular changes in human airway epithelial cells The study found that LKβ1 expression was downregulated in BOS exosomes compared to stable exosomes. LKβ1 knockdown induced a decrease in EMT markers, phosphorylated mTOR, and phosphorylated AMPK in cells, whereas BOS-exosome inhibited LKβ1 expression and induced EMT labeling. In air-liquid interface culture, BOS-exosome treatment reduced LKβ1 expression, and BOS exosomes significantly induced down-regulation of Vimentin and E-calmodulin and induced EMT labeling. Although the study did not further analyze exosomal contents, exosomes as therapeutic targets will be realized sooner rather than later with the rapid development of high-throughput technologies.

Blocking exosome release from donor lungs has the potential to prevent allograft rejection. In a mouse model of Mycobacterium tuberculosis infection, it was found that mice knocked out of the Rab27a gene were less efficient at stimulating T cells because of their reduced ability to release exosomes. There are small inhibitory molecules such as the neutral sphingomyelinase inhibitor GW4869, the acidic sphingomyelinase phospholipase inhibitor promethazine hydrochloride, which effectively block exosome induction from various tissues and cells. However, one study showed that although GW4869 decreased exosome secretion, it enhanced the secretion of more plasma membrane-derived extracellular vesicles (EVs); Rab27a, while decreasing exosome secretion, could also decrease the secretion of some non-EV-binding soluble factors, suggesting that these inhibitory methods may indirectly affect EV composition and secretion and alter cellular functions (72), Meanwhile, in the normal physiological process of the body, exosomes can realize intercellular communication and maintain the stability of the internal environment. Therefore only block the release of exosomes from the transplanted organ and do not affect the release of exosomes from other tissues or cells. Blocking exosome induction in the donor lung during *in vitro* lung perfusion maybe reduce lung injury and improve the function of the transplanted organ so that even marginal lungs can be successfully transplanted. Ravichandran et al. (72) mentioned that it is feasible to block exosome formation and release during lung perfusion *in vitro*.

Immunogenicity of allogeneic or autoantigens depends on the dose, presentation site, other signals and the nature/state of APC activation. The ability of exosomes to induce immune tolerance remains unexplored, but exosomes have recently been investigated as therapeutic delivery vehicles. Studies have demonstrated that exosomes play an important role in maternal tolerance to fetal alloantigens during pregnancy and in immune privilege associated with tolerance to allogeneic liver transplantation in laboratory rodents (87). In intestinal, cardiac, liver, or kidney transplantation models, exosomes in combination with low-dose immunosuppressants or donor-specific Treg can effectively enhance allograft survival (72, 88). Exosomes may open new perspectives for dealing with severe post-transplant side effects and improving allograft survival.

**Figure 4:**
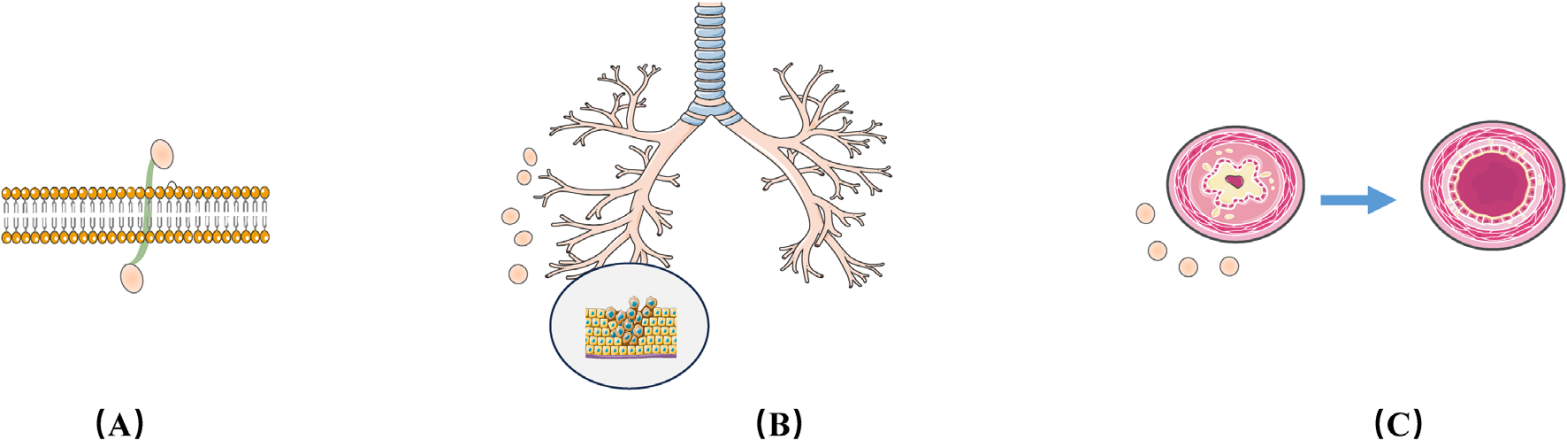
Therapeutic Strategies for Exosomes in LTx Rejection. **(A)** Exosomes have high membrane penetrability, making them excellent drug carriers; **(B)** Exosomes can regulate Epithelial-Mesenchymal Transition (EMT) markers expression in bronchial epithelial cells; **(C)** Exosomes can improve injured bronchial endothelium.

More importantly, exosomes have low toxicity, are not at risk of tumor formation, and because of their small size, easily diffuse across biological barriers, which allows them to be used as injectable therapeutic agents (89–91). The Adipose-Derived Stem Cell exosome miR-125b-5p attenuates Ferroptosis in lung microvascular endothelial cells in septic lung injury via Keap1/Nrf2/GPX4, half of the mice died at 24 h and 80% died at 48 h after Cecum Ligation and Puncture (CLP), After tail vein injection of ADSCs exosomes in mice, mortality decreased to 10% at 24 hours and 60% at 48 hours. Lung tissues were collected for HE staining and scored for injury by Lung Injury Score, Bronchoalveolar Lavage Fluid (BALF), and Wet-Dry Ratio analysis. The HE results showed swollen and congested alveolar capillaries, hemorrhage in the alveolar lumen, and inflammatory cellular infiltration in mice in the sepsis group compared with the sham-operated group. And ADSCs exosomes significantly improved after injury. Furthermore, sepsis increased the lung injury score in mice, whereas ADSCs exosomes decreased the lung injury score. Thus, intravenous injection of ADSCs exosomes attenuated sepsis-induced acute lung injury in mice (92) [as shown in **Figure 4**].

In recent years, in order to overcome the limitations of natural exosomes, artificial exosomes based on nanobiotechnology have emerged. Artificial exosomes with low immunogenicity and toxicity can efficiently transport drugs, proteins and nucleic acids (49, 93, 94), The lipid bilayer structure of exosomes can act as a natural protective barrier for the contents (95), preventing the breakdown of the carrier by surrounding enzymes, and will play an important role in the LTx field.

## 6 Discussion

Although exosomes show great potential in the biomedical field, there is a lack of uniform quality control standards in the isolation, characterization and analysis of exosomes, which limits the promotion of their clinical applications. Existing studies have used different isolation techniques and analytical methods, resulting in significant differences in the purity and functional reproducibility of exosomes (96). For example, the MISEV2018 guidelines proposed by the International Society for Exosomes (ISEV) emphasize the use of standardized isolation and identification methods in exosome studies, but in practice, many studies still do not follow these standards, leading to questionable comparability and reliability of results. Therefore, the development and implementation of rigorous quality control standards is a critical step in advancing the translation of exosome research into the clinic.

In addition, the reproducibility problem in exosome research is mainly affected by a variety of factors, including sample processing, analytical techniques and experimental design. First, different methods used in the isolation and purification of exosomes (e.g., ultracentrifugation, immunocapture, etc.) may lead to differences in the purity and composition of exosomes, thus affecting the reproducibility of experimental results (97). Secondly, technical differences between laboratories and the level of experience of operators can also contribute to the variability of results. For example, using the same exosome analysis technique in different laboratories may produce different results due to factors such as equipment calibration, reagent batch (98). Finally, the lack of adequate experimental records and transparent research methods also makes it difficult for the results of exosome studies to be verified by other researchers. Therefore, the establishment of a systematic experimental process and standardized operating procedures will help to improve the reproducibility of exosome research and promote its development in clinical applications (10, 99, 100).

Exosome-mediated allogeneic recognition involves acquired immunity. Numerous studies have investigated the role of direct, indirect and semi-direct pathways in allogeneic recognition driving lung transplant rejection. Further studies on the role of exosomes of donor or host origin in localized or secondary lymphoid organs in exosome-mediated allogeneic recognition may provide new perspectives for solving outstanding problems in the field of lung transplantation. Clearance of donor permissive leukocytes, or failure to migrate to recipient lymphoid tissues, suggests that donor graft-derived exosomes play an important role in the cognate antigen recognition pathway. These pathways are critical to understanding the development of these immune processes and will provide insights for future therapies and biomarker identification for better patient outcomes. Exosomes are an important biomarker for identifying LTxRs that may be at risk for CLAD. Related studies are also needed to reveal novel mechanisms of exosome biogenesis from lung transplantation and the different roles of exosomes in the development of CLAD after LTx in humans. Exosomes are emerging as effective nanocarriers for drug delivery to target cells and are expected to be a highly promising transport vehicle for development in the field of lung transplantation.
